# Durable Effects of Acupuncture for Knee Osteoarthritis: A Systematic Review and Meta-analysis

**DOI:** 10.1007/s11916-024-01242-6

**Published:** 2024-04-18

**Authors:** He Chen, Hangyu Shi, Shuai Gao, Jiufei Fang, Jiamin Yi, Wenhui Wu, Xinkun Liu, Zhishun Liu

**Affiliations:** 1 Department of Acupuncture, China Academy of Chinese Medical Sciences, Guang’anmen Hospital, No. 5 Beixiange St, Xicheng District, Beijing, 100053 China; 2 Graduate College, Beijing University of Chinese Medicine, Beijing, China; 3 Guangzhou University of Chinese Medicine, Guangzhou, China

**Keywords:** Acupuncture, Knee osteoarthritis, Durable effects, Pain, Function

## Abstract

**Purpose of Review:**

Knee osteoarthritis (KOA) is a degenerative joint disease which can result in chronic pain and disability. The current interventions available for KOA often fail to provide long-lasting effects, highlighting the need for new treatment options that can offer durable benefits. Previous studies have suggested the efficacy of acupuncture for knee osteoarthritis (KOA) with its durability remaining uncertain. In this review, we aimed to investigate the durability of the efficacy after completion of treatment.

**Recent Findings:**

We performed thorough searches of PubMed, EMBASE, Web of Science, and Cochrane Central Register of Controlled Trials from inception to November 4, 2023. The outcomes were assessed at all available time points after completion of treatment. Primary outcomes were changes from baseline in pain and function measured using the Western Ontario and McMaster Universities Osteoarthritis Index (WOMAC) pain and function subscales. Secondary outcomes included response rate, overall pain, the WOMAC stiffness subscale, total WOMAC index, and physical and mental health components of 12/36-item Short-Form Health Survey. A total of 10 randomized controlled trials (RCTs) involving 3221 participants were included. Pooled estimates suggested that acupuncture may offer potential improvements in function and overall pain for 4.5 months post-treatment versus sham acupuncture (SA). Acupuncture may provide durable clinically important pain relief and functional improvement up to 5 months post-treatment versus usual care, and up to 6 months post-treatment versus diclofenac. For acupuncture versus no treatment, one trial with large sample size indicated that improvements in pain and function persisted for 3 months post-treatment, while the other trial reported that significant pain reduction and functional improvement were only observed at the end of the treatment, not at 9 months post-treatment. However, acupuncture as adjunct to exercise-based physical therapy (EPT) showed no superiority to SA as an adjunct to EPT or EPT alone up to 11.25 months after completion of treatment.

**Summary:**

Acupuncture may provide pain alleviation and functional improvements in KOA patients for 3 to 6 months after completion of treatment with a good safety profile.

**Supplementary Information:**

The online version contains supplementary material available at 10.1007/s11916-024-01242-6.

## Introduction

Knee osteoarthritis (KOA) is a degenerative joint disease that results from wear and tear and progressive loss of articular cartilage [1]. This condition causes chronic pain and locomotor disability, leading to significant impact on mobility, quality of life, and livelihood, particularly among the elderly population [2]. Moderate to severe KOA can present with widespread anterior knee pain with distal radiation [3], while persistent nocturnal pain that disrupts sleep or rest occurs in advanced stage [4]. The pooled global prevalence of KOA stands at 22.9% in individuals aged 40 and above [5]. KOA is ranked among the prime contributors to global years lived with disability (YLD) and exacts considerable economic and societal toll, making it a public concern [6, 7]. Given the aging population and escalating obesity rates, this burden is projected to increase [8].

The structural progression of the disease cannot be altered, and thus long-term management should be highlighted. Current non-pharmacological interventions for KOA including therapeutic exercise, weight management, and self-efficacy and self-management programs are strongly recommended [9–12] and form the foundation of management. However, exercise is time-consuming and current evidence is insufficient to recommend specific exercise prescriptions. Moreover, patients who are in pain might be hesitant to engage in exercise, and there is no uniformly accepted threshold of pain indicating whether or not a patient should exercise [9]. The effect sizes of self-efficacy and self-management programs are generally small [9]. Besides, these interventions need to be maintained and long-term adherence to them often proves unsatisfactory [2]. As for pharmacological interventions, topical and oral non-steroidal anti-inflammatory drugs (NSAIDs) are strongly recommended [9–11]. However, NSAIDs are associated with short-lived symptomatic relief, and oral NSAIDs may cause considerable side effects impacting cardiovascular, gastrointestinal, renal, and liver systems [13]. Intra-articular corticosteroid injections are also recommended when other pharmacological treatments are ineffective or unsuitable, but it can only provide short-term relief (2 to 10 weeks) [9–12]. Recent guidelines recommend against [10] or conditionally recommend against [9, 11] intra-articular hyaluronic acid injection due to its minimal pain alleviation and potential increase in adverse events [14]. Moreover, a systematic review included studies of KOA patients with at least 12 months of follow-up, and investigated the association of various pharmacological interventions with long-term pain management; the results suggested an uncertainty surrounding the effect estimates for change in pain for all comparisons with placebo [15]. In addition, genicular nerve block only offers short-term pain relief for approximately six weeks following treatment [16]. Currently, scant and low-quality evidence implies peripheral nerve stimulation may reduce postoperative knee pain in the short term [17, 18]; further studies are warranted to determine its efficacy for knee osteoarthritis [19]. This highlights the need for validated alternative treatment options with durable effects.

Acupuncture is a safe treatment extensively used for chronic pain. Prior research has indicated that acupuncture can significantly alleviate pain and improve physical function and quality of life in KOA patients [20–23]. Durable effects refer to the sustained and long-lasting benefits of treatment beyond the immediate or short-term effects. Currently, evidence concerning the durable effects of acupuncture for KOA remains sparse and inconsistent. Two reviews reported short- and long-term improvements in pain and function after acupuncture treatment compared with SA [24, 25]. One review reported pain alleviation after treatment but no functional improvements versus SA within 1 month post-treatment [26]. Another meta-analysis concluded that acupuncture could provide superior short-term pain control, and short- and long-term function improvements [27]. Given that the definitions of time frames vary and pooled results are limited, we conducted this systematic review and meta-analysis focusing on all available outcomes measured after completion of treatment to investigate the durability of the effects of acupuncture for KOA.

## Methods

### Data Sources

We undertook this systematic review and meta-analysis adhering to the Cochrane Handbook for Systematic Reviews of Interventions version 6.3 [28] and arranged our reports in compliance with the Preferred Reporting Items for Systematic Reviews and Meta-Analyses (PRISMA) guideline [29]. The review was prospectively registered on PROSPERO (CRD42023446663). We only considered studies published in English and did not take Chinese publications into consideration because our preliminary research of Chinese databases found most trials were poorly reported without available post-treatment outcomes. One researcher conducted a systematic search of PubMed, EMBASE, Web of Science, and Cochrane Central Register of Controlled Trials from inception to March 31, 2023. We subsequently updated our search until November 4, 2023. Citation management was performed using Endnote X9 (Clarivate). The search strategy is presented in Table S1 in the supplement.

### Eligibility Criteria

Two researchers independently selected studies and disagreement was resolved by discussion. We screened for published randomized controlled trials (RCTs) of acupuncture in patients aged 18 years and older diagnosed with KOA. Patients who had a history of knee surgery or injury were excluded. We excluded RCT registries or ongoing studies. We included RCTs in which acupuncture involved the insertion of needles into the skin. We also take auricular and abdominal acupuncture and scalp acupuncture into consideration but excluded fire needling, warming needle, and acupoint injection. Manual acupuncture (MA), electro-acupuncture (EA), and dry needling (DN) were all deemed eligible. Eligible types of comparisons included sham acupuncture (SA), usual care, waiting list, and exercise, as well as pharmacological intervention. Studies comparing different types of acupuncture or different acupoint formulas were excluded. Studies lacking outcomes measured at or after 3 months post-treatment or with maintained treatment during follow-up periods were also excluded. We considered trials with small populations (no more than 10 participants per group) ineligible due to potential methodological shortcomings leading to low-certainty evidence.

### Data Extraction

Two researchers independently extracted data from included RCTs using standardized forms via Excel 2019. The following data were extracted: the first author, location, publication year, basic demographics of the participants, sample size, intervention details, key outcomes, follow-up time points, and adverse events. We attempted to contact study authors when data were not readily available.

### Outcomes

Data analysis was performed based on a monthly time frame, wherein a 4-week period was considered equivalent to 1 month and post-treatment was defined as after the final treatment session. The outcomes were assessed at all available time points after completion of treatment. Primary outcomes were changes from baseline in pain and function measured using the WOMAC pain and function subscales. We also assessed whether the effects size estimates of the primary outcomes met the cutoff value for minimal clinically important differences (MCIDs). The MCIDs for KOA have been estimated to be an SMD of 0.39 for WOMAC pain subscale and 0.37 for WOMAC function subscale [30].

The WOMAC is the most widely used and thoroughly validated instrument for assessing patients with KOA [31–34]. It is a 24-item questionnaire for assessing pain (5 items), physical function (17 items), and stiffness (2 items) in individuals with osteoarthritis of the knee and hip. The WOMAC pain subscale evaluates the severity and frequency of pain experienced in the affected joint during various activities. The WOMAC function subscale measures the individual’s ability to carry out specific tasks or activities of daily living, such as walking, climbing stairs, and getting in and out of chairs. The WOMAC stiffness subscale assesses the degree of joint stiffness and how it affects movement and mobility. The WOMAC is available in 5-point Likert, 11-point numerical rating, and 100-mm visual analogue scale (VAS) formats. Higher scores indicate greater severity of pain, stiffness, or difficulty in performing physical activities. The scores from each subscale can be summed to obtain a total score (WOMAC index) or analyzed separately to assess different aspects of osteoarthritis symptoms.

Secondary outcomes include response rate (primarily based on pain and function, the specific definition may vary among trials), and changes from baseline in overall pain (measured by VAS/NRS [numeric rating scale] scores, higher scores indicate greater pain intensity), WOMAC stiffness subscale, and total WOMAC index, and physical and mental health components of 12/36-item Short-Form Health Survey (higher scores indicate better status). For RCTs with more than one intervention group (e.g., EA and MA), we merged the data from each group when synthesizing in one meta-analysis as recommended by the Cochrane Handbook [28].

### Assessment of Risk of Bias

Two researchers independently evaluated the risk of bias of the RCTs using the revised Cochrane risk of bias, version 2 (RoB 2) tool [35]. Disagreements were resolved by consensus. RoB 2 provides a structured approach to evaluate several domains of bias, including randomization process, deviations from intended interventions, missing outcome data, measurement of outcomes, and reporting of study findings. The risk of bias of each domain was categorized into three levels, low, some concerns, or high. The overall risk of bias for the study was deemed high if one or more individual domains were evaluated as having a high risk of bias. The overall risk of bias was categorized as low only when all components received a low risk of bias rating. Otherwise, the risk of bias was judged as some concerns.

### Statistical Analysis

We extracted the mean differences of changes in forementioned outcome measurements and their corresponding standard deviations (SDs) for both intervention and control groups. In cases where variance data were not reported as SDs, we calculated them from the trial data, including sample size and other variance indicators such as standard error (SE) of mean or 95% confidence intervals(CIs) using the formula provided by the Cochrane Handbook [28]. Pooled continuous data were expressed as standardized mean differences (SMDs) with 95% CIs. Pooled dichotomous data were presented as risk ratios (RR) with 95% CIs. For safety outcomes, considering the number of events was generally very low, we calculated odds ratio (OR) instead of RR. The odds ratio greater than 1 suggests a higher probability of events occurring in the acupuncture group versus control. The corresponding *p* value less than 0.05 indicated the estimate was statistically significant. We synthesized data by the random effects model considering the potential heterogeneity of the RCTs’ intervention protocols and settings. *I*^2^ statistics were reported to assess heterogeneity. An *I*^2^ value greater than 50% represented substantial heterogeneity. Publication bias was not assessed due to the limited number of studies included in the quantitative synthesis. All analyses were conducted using the meta package[36] in R (version 4.1.1) [37].

## Results

### Characteristics of Included Studies

**Fig. 1:**
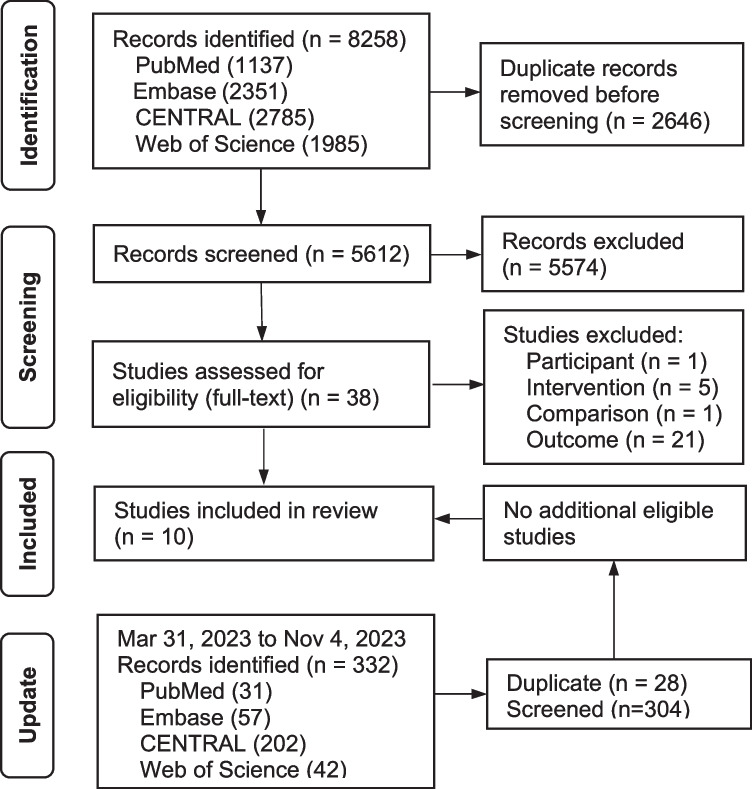
Flow diagram of the identification and selection of studies

A total of 10 RCTs were identified from 8258 citation records and 332 updated records (Fig. 1). Excluded studies with reasons are listed in Table S2. The included studies consisted of 3221 participants (Table 1). These trials were published between 2005 and 2023, with eight of them being multi-centered. The trials were conducted in various countries, including China [23, 38–40], Germany [41, 42], the United States [43], the United Kingdom [44], Spain [45], and Australia [46]. Among these studies, seven trials investigated MA, one both MA and EA, and two DN. The frequency of treatment ranged from 1 to 3 times per week over a period of 4 to 12 weeks, and the duration of the observation ranged from 4 to 12 months. The average age of the participants was mostly over 60. In nine trials, female sex was dominant. Analyses in eight trials were based on the intention-to-treat (ITT) population, while two trials were based on the per-protocol population [40].

**Table 1:** Characteristics of included trials

**Study ID**	**Location** **Center**	**Age mean (SD), y** **Female (%)**	**Intervention**					**Control**		**Duration of observation**	**Measurement time points**
			**Treatment**	**Sessions, n**	**Time/session**	**Duration, weeks**	**N**	**Type**	***N***		
T1 Witt 2005 [41]	Germany Multicenter	64 (6.5) 68.8%	MA	12	At least 30 min	8	149	SA: minimal acupuncture, superficial needling at non-acupuncture points	75	52 weeks	8, 26, 52 (weeks)
T2 Scharf 2006 [42]	Germany Multicenter	62.8 (8.8) 68.8%	MA	10 (+ 5)^*^	20–30 min	6	326	SA: needles inserted superficially away from acupuncture points	365	26 weeks	13, 26 (weeks)
								Conservative therapy: 10 physician visits with consultation and prescription for diclofenac	316		
T3 Foster 2007 [44]	the United Kingdom Multicenter	63.2 (8.8) 58.2%	Advice, exercise (6 wk) + MA	6	25–35 min	3	117	Advice, exercise (6 wk) + SA (non-penetrating)	119	12 months	2 weeks, 6 weeks, 6 months, 12 months
								Advice and exercise	116		
T4 Chen 2013 [43]	the United States Multicenter	60.5 (11.4) 51.6%	MA	12	20 min	12	104	SA: non-penetrating	109	26 weeks	12 weeks, 26 weeks
T5 Hinman 2014 [46]	Australia Single center	63.5 (8.7) 51.1%	MA	8–12	20 min	12	70	Control: no treatment	71	1 year	12 weeks, 1 year
T6 Lin 2018 [38]	China Multicenter	59.8 (7.3) 88.1%	MA	24	20 min	8	21	SA: needles were inserted vertically about 3–5 mm into non-acupoints without manipulation	21	26 weeks	8, 16, 26 (weeks)
T7 Tu 2021 [23]	China Multicenter	62.8 (7.1) 76%	EA	24	30 min	8	151	SA: 8 non-acupoints were superficially penetrated (2–3 mm) without needle manipulation for de qi	146	26 weeks	4, 8, 16, 26 (weeks)
			MA	24	30 min	8	145				
T8 Liu 2022 [39]	China Multicenter	60.7 (8.8) 80.3%	MA at acupoints with lower pain threshold	12	30 min	4	221	Waiting-list group: non-acupuncture treatments were allowed upon requested	221	16 weeks	4, 8, 12, 16 (weeks)
			MA at acupoints with higher pain threshold	12	30 min	4	219				
T9 Sánchez Romero 2020 [45]	Spain Multicenter	72.3 (5.7) 29%	DN + exercise	6	NR	6	31	SA (non-penetrating) + exercise	31	12 months	3, 6, 9, 12 months
T10 Ma 2023 [40]	China Single center	75 (6.1) 67.5%	DN	≤ 6	NR	6	42	Diclofenac sodium	35	6 weeks and 6 months	6 weeks after treatment, 6 months after treatment

*N* number of participants, *SA* sham acupuncture, *EPT* exercise-based physical therapy, *EA* electro-acupuncture, *MA* manual acupuncture, *DN* dry needling, *min* minute, *NR* not reported

*5 additional acupuncture sessions were administered if the treatment was graded as partially successful after 6 weeks

The outcome measures employed in the trials are documented in Table S3. It should be noted that the definition of response rate varied among trials. In the trials by Scharf et al. and Chen et al., response rate was defined as at least 36% improvement in total WOMAC index [47, 48]. Foster et al. defined the response rate according to criteria from the outcome measures in Rheumatology and Osteoarthritis Research Society international initiative (OMERACT-OARSI) [49, 50], while Tu and colleagues defined the response rate as the proportion of participants who simultaneously achieved minimal clinically important improvement on the NRS (2 points on the 11-point scale) and WOMAC function (6 points on the 68-point scale) subscales.

The details of the treatment are presented in Table S4. All the acupuncturists delivering treatments were qualified and experienced. Only one trial clearly reported that all acupuncturists involved were trained in standardized operating procedures before the start of the project [23]. Eight trials based on meridian and collateral theory selected a combination of local and distal acupoints. Moreover, the most commonly selected acupoints included ST36 (Zusanli), GB34 (Yanglingquan), ST35 (Dubi), SP9 (Yinlingquan), SP10 (Xuehai), SP6 (Sanyinjiao), ST34 (Liangqiu), LR8 (Ququan), BL60 (Kunlun), KI10 (Yingu), and BL40 (Weizhong).

### Rob Assessment

**Fig. 2:**
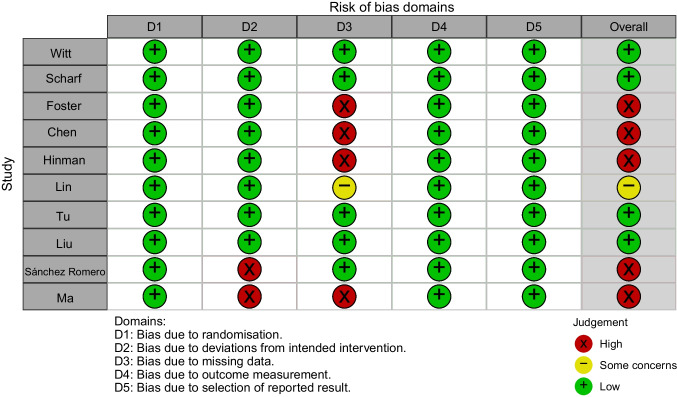
Risk of bias summary

The assessment of RoB 2 indicated high risk for five trials [40, 43–46], some concerns for one trial [38], and low risk for four trials [23, 39, 41, 42] (Fig. 2, Fig. S1). The domain of missing outcome data was of most concern. Two studies performed per-protocol analysis [40, 45], which may lead to potential impact on the result. Therefore, they were judged as of high risk of bias in the domain of deviations from intended intervention. Four studies were rated as of high risk of bias in the domain of missing outcome data [40, 43, 44, 46]. These four studies had more than 5% of loss to follow up and provided no evidence to judge whether the result was biased by missing outcome data or not. The reasons for withdrawal and no questionnaire in the study by Foster et al. [44] were not documented in detail. In the other three studies [40, 43, 46], the reasons for withdrawal were not balanced between groups. The study by Lin et al. [38] was rated as of some concern in the domain of missing outcome data due to more than 5% of loss to follow up but the number of participants lost to follow up and the reasons were generally balanced between the two groups.

### Acupuncture Versus SA

**Fig. 3:**
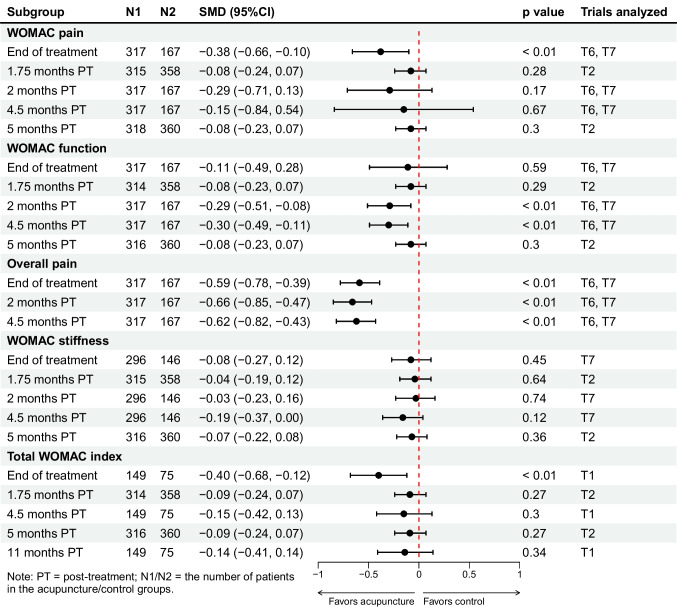
Acupuncture versus sham acupuncture: changes from baseline in WOMAC pain, WOMAC function, overall pain, WOMAC stiffness, and total WOMAC index

Four trials enrolling 1399 participants compared acupuncture with SA [23, 38, 41, 42]. Pooled estimates suggested that acupuncture may offer potential improvements in function and overall pain for 4.5 months after completion of treatment. For primary outcomes, acupuncture was superior to SA in pain reduction only at the end of the treatment (SMD − 0.38, 95%CI − 0.66 to − 0.10) (Fig. 3). For WOMAC function subscale, acupuncture provided significant improvement in function at 2 months (SMD − 0.29, 95%CI − 0.51 to − 0.08) and 4.5 months (SMD − 0.30, 95%CI − 0.49 to − 0.11) after completion of treatment (*p* < 0.05), while no significant improvement was observed at other time points (Fig. 3).

**Fig. 4:**
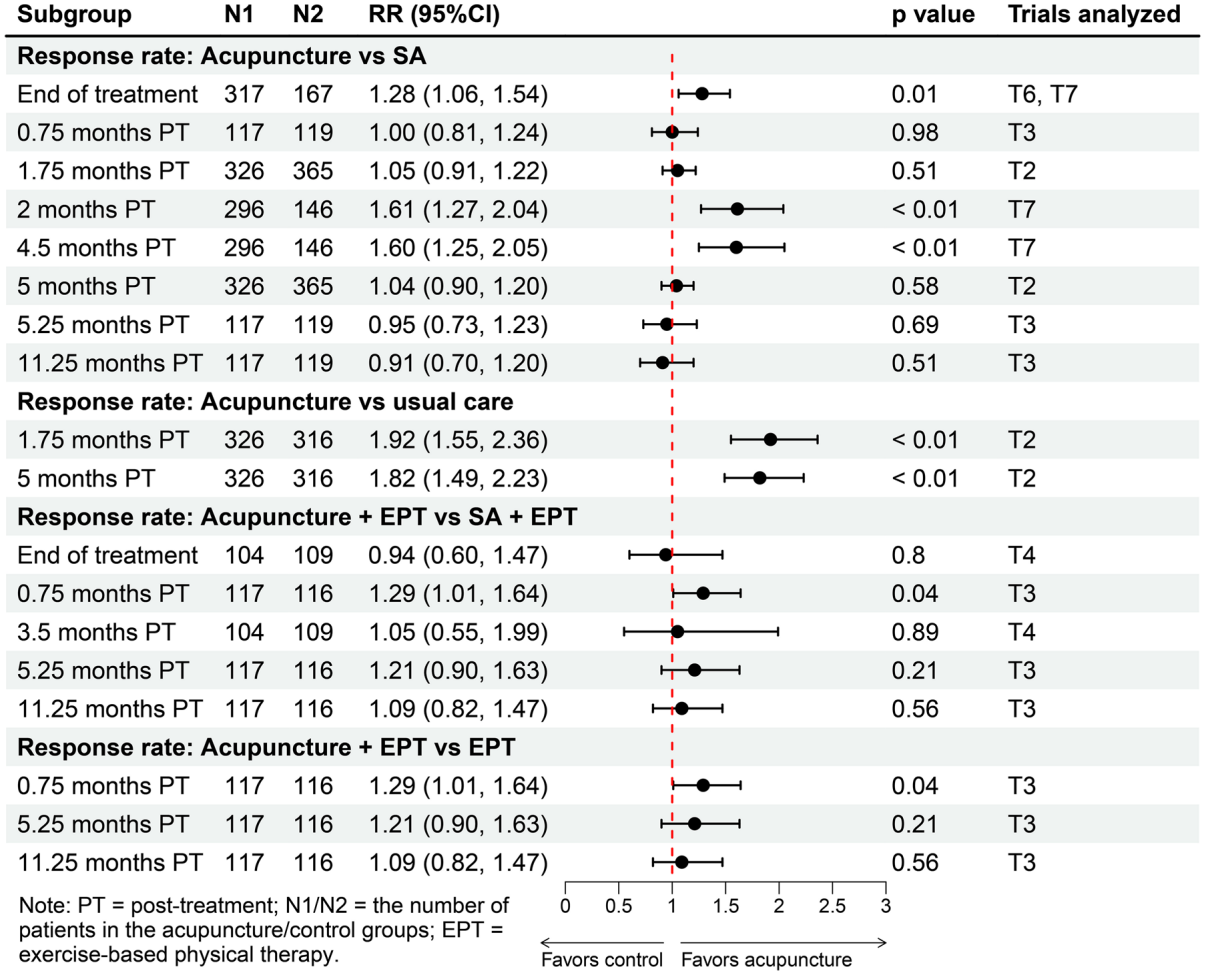
Response rate

Regarding the secondary outcomes, patients receiving acupuncture reported significant greater reduction in overall pain (*p* < 0.05) comparing with patients receiving SA at the end of the treatment (SMD − 0.59, 95%CI − 0.78 to − 0.39), and at 2 months (SMD − 0.66, 95%CI − 0.85 to − 0.47) and 4.5 months (SMD − 0.62, 95%CI − 0.82 to − 0.43) post-treatment (Fig. 3). Acupuncture provided no improvement versus SA in WOMAC stiffness subscale up to 10.5 months post-treatment, and significant improvement in total WOMAC index was only found at the end of the treatment (SMD − 0.40, 95%CI − 0.68 to − 0.12) (Fig. 3). In terms of quality of life, results showed that acupuncture significantly improved physical (SMD 0.24, 95%CI 0.05 to 0.42) and mental (SMD 0.21, 95%CI 0.02 to 0.40) health only at 2 months post-treatment (Fig. S2). In addition, there were significantly higher response rates in the acupuncture group at the end of the treatment (RR 1.28, 95%CI 1.06 to 1.54), and at 2 months (RR 1.61, 95%CI 1.27 to 2.04) and 4.5 months (RR 1.60, 95%CI 1.25 to 2.05) after completion of treatment (Fig. 4).

### Acupuncture Versus Usual Care

**Fig. 5:**
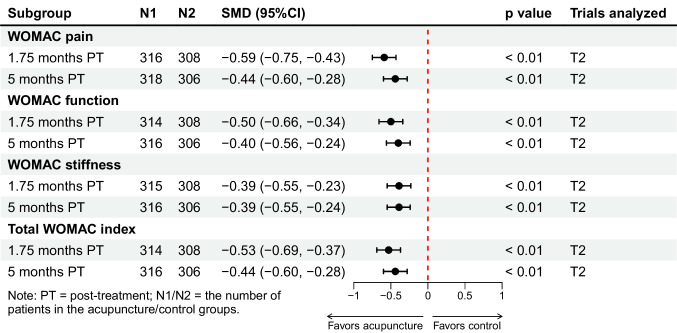
Acupuncture versus usual care: changes from baseline in WOMAC pain, WOMAC function, overall pain, WOMAC stiffness, and total WOMAC index

We categorized the conservative group in the trial by Scharf et al. [42] as usual care. Results showed that acupuncture may offer sustainable benefits for KOA patients up to 5 months after completion of treatment compared with usual care. For primary outcomes, acupuncture provided significant (*p* < 0.05) and clinically important improvements in pain at 1.75 months (SMD − 0.59, 95%CI − 0.75 to − 0.43) and 5 months (SMD − 0.44, 95%CI − 0.60 to − 0.28) post-treatment, and in function at 1.75 months (SMD − 0.50, 95%CI − 0.66 to − 0.34) and 5 months (SMD − 0.40, 95%CI − 0.56 to − 0.24) post-treatment (Fig. 5). For secondary outcomes, acupuncture provided significant improvements in WOMAC stiffness subscale, total WOMAC index, and physical health up to 5 months after treatment termination (Fig. 5, S3). No significant improvement in mental health was observed (Fig. S3). Moreover, the acupuncture group had a significantly higher response rate compared with the usual care group up to 5 months post-treatment (Fig. 4).

### Acupuncture Versus No Treatment

The waiting-list group in the trial by Liu et al. [40] and the control group in the trial by Hinman et al. [46] were categorized as no treatment controls. Liu and colleagues enrolled 661 participants and reported that acupuncture resulted in significant improvements in pain and function, as well as significant reduction in WOMAC stiffness and total WOMAC index up to 3 months after completion of treatment [40] (Fig. S4). In the trial by Hinman et al. [46], primary outcomes were overall pain measured using an 11-point NRS and function measured using the WOMAC function subscale (range 0 to 68 points). Analyses based on ITT population (141 participants) showed that acupuncture resulted in modest improvements in pain (MD [mean difference] − 1.1, 95% CI − 1.8 to − 0.4) at the end of the treatment, but not at 9 months post-treatment. Acupuncture resulted in modest improvement in function compared with no treatment control at the end of the treatment (MD − 3.9, 95% CI − 7.7 to − 0.2) but was not maintained at 9 months post-treatment. In addition, acupuncture provided borderline significant reduction in the WOMAC pain subscale (range 0–20) at the end of the treatment (MD − 1.2, 95%CI − 2.3 to 0.0, *p* = 0.05) and at 9 months (MD − 1.4, 95%CI − 2.7 to 0.0, *p* = 0.05) post-treatment.

### DN Versus Diclofenac

**Fig. 6:**
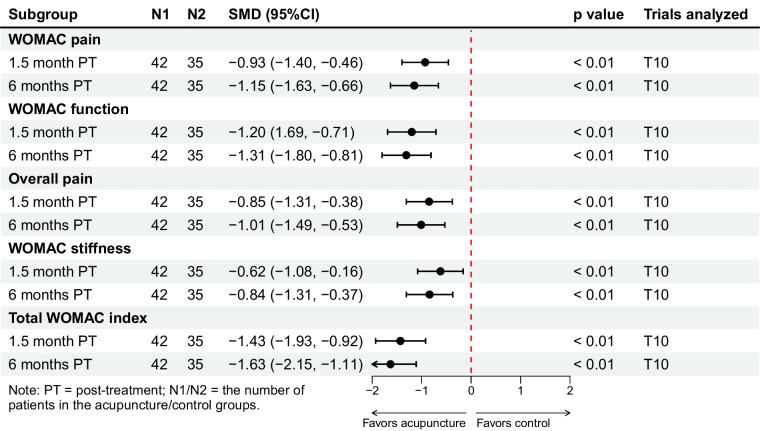
Dry needling versus diclofenac: changes from baseline in WOMAC pain, WOMAC function, overall pain, WOMAC stiffness, and total WOMAC index

One trial enrolling 77 participants compared DN with diclofenac [40]. Analyses based on per-protocol population [40] showed that DN appeared to provide significant and clinically important improvements in pain and function, as well as significant improvements in overall pain, WOMAC stiffness subscale, and total WOMAC index up to 6 months after completion of treatment (*p* < 0.05) (Fig. 6).

### Acupuncture Plus EPT Versus SA Plus EPT or EPT Alone

Three trials involving 511 patients compared acupuncture as adjunct to EPT with SA as an adjunct to EPT [43–45]. Overall, pooled data showed no association favoring acupuncture as adjunct to EPT in pain, function, overall pain, WOMAC stiffness subscale, total WOMAC index, and physical and mental health up to 11.25 months post-treatment (Fig. S5). Regarding response rate, higher response rate was only observed at 0.75 months post-treatment (RR 1.29, 95%CI 1.01 to 1.64) (Fig. 4). For acupuncture plus EPT versus EPT, a significant reduction in overall pain (SMD − 0.37, 95%CI − 0.63 to − 0.11) and a higher response rate (RR 1.29, 95%CI 1.01, 1.64) were observed at 0.75 months post-treatment (*p* < 0.05), while other results were insignificant (Fig. S5, 4).

### Adverse Events

In ten included trials, one [45] did not report adverse events. All adverse events related to acupuncture were mild and transient, primarily needling pain and hematoma. Pooled data of the number of patients reporting adverse events indicated that acupuncture was a safe treatment modality (OR 0.98, 95%CI 0.76 to 1.26, *p* = 0.88; 3 trials, 1699 participants) (Fig. S7).

## Discussion

In this present study, we synthesized data measured at the same time points, which allowed us to analyze the durable effects of the intervention in a more precise and standardized manner. For acupuncture versus SA, results suggested that acupuncture offered potential improvements in function and overall pain for 4.5 months after completion of treatment. Acupuncture may provide durable clinically important pain relief and functional improvement up to 5 months post-treatment compared with usual care, and up to 6 months post-treatment compared with diclofenac. For acupuncture versus no treatment, one trial with large sample size indicated improvements in pain and function persisting for 3 months post-treatment, but the other reported that significant pain reduction and functional improvement were only observed at the end of the treatment, not at 9 months post-treatment. However, acupuncture as adjunct to EPT showed no superiority to SA as an adjunct to EPT or EPT alone up to 11.25 months after completion of treatment.

Our results revealed the potential sustainable improvements in pain and function of acupuncture for KOA after completion of treatment, which is consistent with an individual participant meta-analysis that reported acupuncture was superior to SA for multiple pain conditions including osteoarthritis with the effect appearing to persist for 12 months, and the random effects estimates of pain reduction of acupuncture for osteoarthritis were 0.74 (SMD, 95%CI 0.46 to 1.01) versus no-acupuncture control and 0.45 (SMD, 95%CI 0.15 to 0.75) versus SA [51].

Additionally, it is illustrative to compare our results with those of other interventions routinely used in clinical practice. A meta-analysis of oral NSAIDs compared with placebo included 23 trials involving 10,845 patients with KOA and found that the effect size for pain reduction was 0.32 (95%CI 0.24 to 0.39) with significant heterogeneity in a random effects model at 2–13 weeks after the initiation of intervention based on WOMAC pain subscale, visual analogue scales (VAS), or other pain scores [52]. Data from ten trials that did not exclude non-responders to NSAID revealed a homogeneous effect estimate for pain reduction of 0.23 (95%CI 0.15 to 0.31) [52]. In 11 trials with 7433 patients, the effect size of reduction in functional disability was 0.29 (95%CI 0.18 to 0.40) with significant heterogeneity at 2–13 weeks after the initiation of intervention [52]. Moreover, one trial reported no significant difference between NSAIDs and placebo in long-term pain reduction at 1 to 4 years after treatment initiation [53]. As short-lived symptomatic relief and considerable side effects are associated with NSAIDs, only limited use was recommended. By contrast, acupuncture may provide durable and clinically important effects with a satisfactory safety profile, making it a promising treatment option. Moreover, Sánchez Romero et al. reported that 90.3% of the patients who received DN had a reduction in analgesic use compared to only 26.3% in the sham DN group [45].

There were two trials of DN with small sample size included in our review. One trial concluded that the combination of DN and exercise program resulted in no reduction in pain or disability versus SA combined with exercise in patients with KOA, while the other trial showed that DN provided significant benefits persisting for 6 months post-treatment. However, both trials were based on per-protocol analysis and had potential methodological shortcomings, which limit the representativeness of the results. Additionally, a prior meta-analysis also found contradictory results for myofascial trigger point dry needling [54]. Another meta-analysis including two trials[45, 55] observed no significant effects at short- (0–10 weeks), mid- (10–20 weeks) and long- (> 20 weeks) term follow-ups post-intervention [56]. Given the limited and varied nature of the available trials, further investigation of DN for KOA is warranted, preferably through high-quality RCTs with long-term follow-up.

In KOA patients, acupuncture may help reduce inflammation, improve blood flow, and release endogenous neurotransmitters by activating autonomic nervous system to help relieve the pain. The central nervous system may integrate afferent signals and regulate the somatosensory autonomic reflexes to produce anti-inflammatory and analgesic effects. In this present study, we found the potential of acupuncture to offer sustained benefits for KOA patients. It may alleviate pain and improve joint function even after the treatment sessions have ended, allowing for ongoing management of the disease and reducing the need for frequent interventions or referrals to specialists. Acupuncture, as a non-pharmacological method, can be a preferred option for individuals who wish to avoid or minimize the use of medications, or in cases where pharmacological interventions are not recommended. Besides, it may also alleviate pressure on the healthcare system and prevent medication addiction or overuse, as well as provide economic benefits due to its cost-effectiveness [57–59].

Notably, our findings should be interpreted as exploratory due to limited trials included and potential methodological shortcomings. This study has several limitations. First, RCT registries or ongoing studies were not considered, and only English-language publications were included. Second, the RoB 2 assessment requires subjective judgment, which may differ across individuals. Third, some of the data were calculated using mathematical formulas recommended by the Cochrane handbook instead of being directly extracted from the reports, and thus the results should be interpreted with caution. Furthermore, the majority of outcomes were measured by self-reported questionnaires, and there still lacks RCTs with long-term follow-up. Therefore, we suggest that future trials of acupuncture for KOA measure both performance and self-reported outcomes and conduct long-term follow-up.

## Conclusion

This systematic review and meta-analysis suggested that acupuncture may offer improvements in pain and function in KOA patients for 3 to 6 months after completion of treatment with a good safety profile. Offering acupuncture to patients may facilitate the ongoing management of KOA. Further studies are warranted to update the evidence and provide definitive conclusions.

### Supplementary Information

Below is the link to the electronic supplementary material.

Supplementary file1 (DOCX 3309 KB)

## Data Availability

All the data generated in this review are available upon request.
