# Supplementary material for: Durable Effects of Acupuncture for Knee Osteoarthritis: A Systematic Review and Meta-analysis
Source: Curr Pain Headache Rep. 2024 Apr 18;28(7):709–22. doi: 10.1007/s11916-024-01242-6 (PMC11271378; doi:10.1007/s11916-024-01242-6)
Supplement: Supplementary file 1 — Supplementary file1 (DOCX 3309 KB) [file 11916_2024_1242_MOESM1_ESM.docx]

**Table S1. Search strategies**

| **PubMed** | **Records** | **Update** |
| --- | --- | --- |
| ("osteoarthritis, knee"[MeSH Terms]) OR Knee Osteoarthritis[Title/Abstract] OR Knee Osteoarthritides[Title/Abstract] OR Osteoarthritis of Knee[Title/Abstract] OR Osteoarthritis of the Knee[Title/Abstract] OR Knee pain[Title/Abstract] OR Arthritis[Title/Abstract] OR joint pain[Title/Abstract] OR gonarthritis[Title/Abstract] OR chronic knee pain[Title/Abstract] OR Osteoarthritis[Title/Abstract] OR Osteoarthritides[Title/Abstract] OR osteoarthroses[Title/Abstract] OR Degenerative arthritis[Title/Abstract] OR Degenerative Arthritides[Title/Abstract] OR Osteoarthrosis Deformans[Title/Abstract] OR Arthrosis[Title/Abstract] OR Arthroses[Title/Abstract]) AND (Acupuncture [MeSH Terms] OR Acupuncture[Title/Abstract] OR Acupuncture therapy[Title/Abstract] OR Acupuncture Treatment[Title/Abstract] OR Electroacupuncture[Title/Abstract] OR Electro-acupuncture[Title/Abstract] OR Electric Stimulation [Title/Abstract] OR Needle[Title/Abstract] OR Needling[Title/Abstract] OR dry needling[MeSH Terms] OR Trigger point[Title/Abstract] OR Trigger point acupuncture[Title/Abstract] OR myofascial trigger point[Title/Abstract]) AND ((randomized controlled trial[pt] OR controlled clinical trial[pt] OR randomized[tiab] OR randomised[tiab] OR placebo[tiab] OR drug therapy[sh] OR randomly[tiab] OR trial[tiab] OR groups[tiab]) NOT (animals[mh] NOT humans[mh])) | **1137** | **31** |
| **EMBASE** | **Records** |  |
| 1. 'knee osteoarthritis'/exp | 44,069 |  |
| 2. ‘Knee Osteoarthritis’:ab,ti OR ‘Knee Osteoarthritides’:ab,ti OR ‘Osteoarthritis of Knee’:ab,ti OR ‘Osteoarthritis of the Knee’:ab,ti OR ‘Knee pain’:ab,ti OR ‘Arthritis’:ab,ti OR ‘joint pain’:ab,ti OR ‘gonarthritis’:ab,ti OR ‘chronic knee pain’:ab,ti OR ‘Osteoarthritis’:ab,ti OR ‘Osteoarthritides’:ab,ti OR ‘osteoarthroses’:ab,ti OR ‘Degenerative arthritis’:ab,ti OR ‘Degenerative Arthritides’:ab,ti OR ‘Osteoarthrosis Deformans’:ab,ti OR ‘Arthrosis’:ab,ti OR ‘Arthroses’:ab,ti | 422,851 |  |
| 3. 'Acupuncture'/exp | 56,491 |  |
| 4. ‘Acupuncture’:ab,ti OR ‘Acupuncture therapy’:ab,ti OR ‘Acupuncture Treatment’:ab,ti OR ‘Electroacupuncture’:ab,ti OR ‘Electro-acupuncture’:ab,ti OR ‘Electric Stimulation’:ab,ti OR ‘Needle’:ab,ti OR ‘Needling’:ab,ti OR ‘dry needling’:ab,ti OR ‘Trigger point’:ab,ti OR ‘Trigger point acupuncture’:ab,ti OR ‘myofascial trigger point’:ab,ti | 220,942 |  |
| 5. 'randomized controlled trial'/exp OR 'controlled clinical trial'/exp OR randomized:ti,ab OR placebo:ti,ab OR 'drug therapy':lnk OR randomly:ti,ab OR trial:ti,ab OR groups:ti,ab | 8,841,809 |  |
| 6. (#1 OR #2) AND (#3 OR #4) AND #5 | **2351** | **57** |
| **The Cochrane Central Registry of Controlled Trials** | **Records** |  |
| 1. MeSH descriptor: [Osteoarthritis, Knee] explode all trees | 5940 | **202** |
| 2. (Knee Osteoarthritis OR Knee Osteoarthritides OR Osteoarthritis of Knee OR Osteoarthritis of the Knee OR Knee pain OR Arthritis OR joint pain OR gonarthritis OR chronic knee pain OR Osteoarthritis OR Osteoarthritides OR osteoarthroses OR Degenerative arthritis OR Degenerative Arthritides OR Osteoarthrosis Deformans OR Arthrosis OR Arthroses):ab,ti,kw | 60,388 |  |
| 3. MeSH descriptor: [Acupuncture] explode all trees | 712 |  |
| 4. (Acupuncture OR Acupuncture therapy OR Acupuncture Treatment OR Electroacupuncture OR Electro-acupuncture OR Electric Stimulation OR Needle OR Needling OR dry needling OR Trigger point OR Trigger point acupuncture OR myofascial trigger point):ab,ti,kw | 42,807 |  |
| 5. (#1 OR #2) AND (#3 OR #4) | **2785** |  |
| **Web of Science** | **Records** |  |
| 1. TS=(Osteoarthritis, Knee OR Knee Osteoarthritis OR Knee Osteoarthritides OR Osteoarthritis of Knee OR Osteoarthritis of the Knee OR Knee pain OR Arthritis OR joint pain OR gonarthritis OR chronic knee pain OR Osteoarthritis OR Osteoarthritides OR osteoarthroses OR Degenerative arthritis OR Degenerative Arthritides OR Osteoarthrosis Deformans OR Arthrosis OR Arthroses) | 380,802 | **42** |
| 2. TS=(Acupuncture OR Acupuncture therapy OR Acupuncture Treatment OR Electroacupuncture OR Electro-acupuncture OR Electric Stimulation OR Needle OR Needling OR dry needling OR Trigger point OR Trigger point acupuncture OR myofascial trigger point) | 195,950 |  |
| 3. TS=((randomized controlled trial OR controlled clinical trial OR randomized OR randomised OR placebo OR drug therapy OR randomly OR trial OR groups) NOT (animals NOT humans)) | 6,068,851 |  |
| 4. #1 AND #2 AND #3 | **1,985** |  |

**Table S2. Excluded studies with reasons**

| **No.** | **Study** | **Reason** | **Explanation** |
| --- | --- | --- | --- |
| **Acupuncture** | | | |
| 1 | Christensen 1992^[1]^ | Outcome | Outcomes were measured at week 3, 5, 6 and 7. No outcome measured after 12 weeks post-treatment. |
| 2 | Takeda 1994^[2]^ | Outcome | No outcome measured after 12 weeks post-treatment. |
| 3 | Berman 1999^[3]^ | Outcome | No outcome measured after 12 weeks post-treatment. |
| 4 | Sangdee 2002^[4]^ | Outcome | Outcomes were measured at week 4.d No outcome measured after 12 weeks post-treatment. |
| 5 | Berman 2004^[5]^ | Intervention | The true acupuncture group underwent 26 weeks of gradually tapering treatment. |
| 6 | Vas 2004^[6]^ | Outcome | No outcome measured after 12 weeks post-treatment. |
| 7 | Witt 2006^[7]^ | Intervention | Patients in the control group received delayed acupuncture treatment (starting 3 months after baseline). |
| 8 | Weiner 2007^[8]^ | Intervention | Periosteal stimulation therapy. |
| 9 | Williamson 2007^[9]^ | Outcome | No outcome measured after 12 weeks post-treatment. |
| 10 | Jubb 2008^[10]^ | Outcome | No outcome measured after 12 weeks post-treatment. |
| 11 | Lansdown 2009^[11]^ | Intervention | Auxiliary treatments of moxibustion and acupressure massage were provided in the experiment group. |
| 12 | Suarez-Almazor 2010^[12]^ | Outcome | No outcome measured after 12 weeks post-treatment. |
| 13 | Lev-Ari 2011^[13]^ | Outcome | The duration of the study is 12 weeks. No outcome measured after 12 weeks post-treatment. |
| 14 | Mavrommatis 2012^[14]^ | Outcome | No outcome measured after 12 weeks post-treatment. |
| 15 | Soni 2012^[15]^ | Outcome | The duration of the study is 12 weeks. No outcome measured after 12 weeks post-treatment. |
| 16 | White 2012^[16]^ | Outcome | No outcome measured after 12 weeks post-treatment. |
| 17 | Ashraf 2014^[17]^ | Outcome | Outcomes were assessed before and after each intervention. No outcome measured after 12 weeks post-treatment. |
| 18 | Elbadawy 2017^[18]^ | Intervention | Periosteal stimulation therapy plus home exercise program versus transcutaneous electrical nerve stimulation |
| 19 | Zhang 2019^[19]^ | Outcome | No outcome measured after 12 weeks post-treatment. |
| 20 | Atalay 2021^[20]^ | Outcome | No outcome measured after 12 weeks post-treatment. |
| 21 | Lam 2021^[21]^ | Outcome | No outcome measured after 12 weeks post-treatment. |
| Dry needling | | | |
| 22 | Itoh 2008^[22]^ | Participant | There were 10 participants in each group. We consider it inadequate if there were no more than 10 participants in each group. |
| 23 | Dunning 2018^[23]^ | Outcome | No outcome measured after 12 weeks post-treatment. |
| 24 | DN Sánchez-Romero 2018^[24]^ | Participant  Outcome | There were 10 participants in each group. No outcome measured after 12 weeks post-treatment. |
| 25 | Farazdaghi 2021^[25]^ | Outcome | No outcome measured after 12 weeks post-treatment. |
| 26 | Amani 2022^[26]^ | Control | A one-armed preliminary study, no control group. |
| 27 | Pang 2022^[27]^ | Outcome | No outcome measured after 12 weeks post-treatment. |
| 28 | Wang 2021^[28]^ | Outcome | No outcome measured after 12 weeks post-treatment. |

**References**

[1] Christensen B V, Iuhl I U, Vilbek H, et al. Acupuncture treatment of severe knee osteoarthrosis. A long-term study [J]. Acta anaesthesiologica Scandinavica, 1992, 36(6): 519-25.

[2] Takeda W, Wessel J. Acupuncture for the treatment of pain of osteoarthritic knees [J]. Arthritis care and research : the official journal of the Arthritis Health Professions Association, 1994, 7(3): 118-22.

[3] Berman B M, Singh B B, Lao L, et al. A randomized trial of acupuncture as an adjunctive therapy in osteoarthritis of the knee [J]. Rheumatology (Oxford, England), 1999, 38(4): 346-54.

[4] Sangdee C, Teekachunhatean S, Sananpanich K, et al. Electroacupuncture versus diclofenac in symptomatic treatment of osteoarthritis of the knee: a randomized controlled trial [J]. BMC complementary and alternative medicine, 2002, 2: 3.

[5] Berman B M, Lao L, Langenberg P, et al. Effectiveness of acupuncture as adjunctive therapy in osteoarthritis of the knee: a randomized, controlled trial [J]. Annals of internal medicine, 2004, 141(12): 901-10.

[6] Vas J, Méndez C, Perea-Milla E, et al. Acupuncture as a complementary therapy to the pharmacological treatment of osteoarthritis of the knee: randomised controlled trial [J]. BMJ (Clinical research ed), 2004, 329(7476): 1216.

[7] Witt C M, Jena S, Brinkhaus B, et al. Acupuncture in patients with osteoarthritis of the knee or hip: a randomized, controlled trial with an additional nonrandomized arm [J]. Arthritis and rheumatism, 2006, 54(11): 3485-93.

[8] Weiner D K, Rudy T E, Morone N, et al. Efficacy of periosteal stimulation therapy for the treatment of osteoarthritis-associated chronic knee pain: an initial controlled clinical trial [J]. Journal of the American Geriatrics Society, 2007, 55(10): 1541-7.

[9] Williamson L, Wyatt M R, Yein K, et al. Severe knee osteoarthritis: a randomized controlled trial of acupuncture, physiotherapy (supervised exercise) and standard management for patients awaiting knee replacement [J]. Rheumatology (Oxford, England), 2007, 46(9): 1445-9.

[10] Jubb R W, Tukmachi E S, Jones P W, et al. A blinded randomised trial of acupuncture (manual and electroacupuncture) compared with a non-penetrating sham for the symptoms of osteoarthritis of the knee [J]. Acupuncture in medicine : journal of the British Medical Acupuncture Society, 2008, 26(2): 69-78.

[11] Lansdown H, Howard K, Brealey S, et al. Acupuncture for pain and osteoarthritis of the knee: a pilot study for an open parallel-arm randomised controlled trial [J]. BMC musculoskeletal disorders, 2009, 10: 130.

[12] Suarez-Almazor M E, Looney C, Liu Y, et al. A randomized controlled trial of acupuncture for osteoarthritis of the knee: effects of patient-provider communication [J]. Arthritis care & research, 2010, 62(9): 1229-36.

[13] Miller E, Maimon Y, Rosenblatt Y, et al. Delayed Effect of Acupuncture Treatment in OA of the Knee: A Blinded, Randomized, Controlled Trial [J]. Evidence-based complementary and alternative medicine : eCAM, 2011, 2011: 792975.

[14] Mavrommatis C I, Argyra E, Vadalouka A, et al. Acupuncture as an adjunctive therapy to pharmacological treatment in patients with chronic pain due to osteoarthritis of the knee: a 3-armed, randomized, placebo-controlled trial [J]. Pain, 2012, 153(8): 1720-6.

[15] Soni A, Joshi A, Mudge N, et al. Supervised exercise plus acupuncture for moderate to severe knee osteoarthritis: a small randomised controlled trial [J]. Acupuncture in medicine : journal of the British Medical Acupuncture Society, 2012, 30(3): 176-81.

[16] White P, Bishop F L, Prescott P, et al. Practice, practitioner, or placebo? A multifactorial, mixed-methods randomized controlled trial of acupuncture [J]. Pain, 2012, 153(2): 455-62.

[17] Ashraf A, Zarei F, Hadianfard M J, et al. Comparison the effect of lateral wedge insole and acupuncture in medial compartment knee osteoarthritis: a randomized controlled trial [J]. The Knee, 2014, 21(2): 439-44.

[18] Elbadawy M A. Effectiveness of Periosteal Stimulation Therapy and Home Exercise Program in the Rehabilitation of Patients With Advanced Knee Osteoarthritis [J]. The Clinical journal of pain, 2017, 33(3): 254-63.

[19] Zhang L, Yuan H, Zhang L, et al. Effect of acupuncture therapies combined with usual medical care on knee osteoarthritis [J]. Journal of traditional Chinese medicine = Chung i tsa chih ying wen pan, 2019, 39(1): 103-10.

[20] Atalay S G, Durmus A, Gezginaslan Ö. The Effect of Acupuncture and Physiotherapy on Patients with Knee Osteoarthritis: A Randomized Controlled Study [J]. Pain physician, 2021, 24(3): E269-e78.

[21] Lam W C, Au K Y, Qin Z, et al. Superficial Needling Acupuncture vs Sham Acupuncture for Knee Osteoarthritis: A Randomized Controlled Trial [J]. The American journal of medicine, 2021, 134(10): 1286-94.e2.

[22] Itoh K, Hirota S, Katsumi Y, et al. Trigger point acupuncture for treatment of knee osteoarthritis--a preliminary RCT for a pragmatic trial [J]. Acupuncture in medicine: journal of the British Medical Acupuncture Society, 2008, 26(1): 17-26.

[23] Dunning J, Butts R, Young I, et al. Periosteal Electrical Dry Needling as an Adjunct to Exercise and Manual Therapy for Knee Osteoarthritis: A Multicenter Randomized Clinical Trial [J]. The Clinical journal of pain, 2018, 34(12): 1149-58.

[24] Sánchez-Romero E A, Pecos-Martín D, Calvo-Lobo C, et al. Effects of dry needling in an exercise program for older adults with knee osteoarthritis: A pilot clinical trial [J]. Medicine, 2018, 97(26): e11255.

[25] Farazdaghi M, Kordi Yoosefinejad A, Abdollahian N, et al. Dry needling trigger points around knee and hip joints improves function in patients with mild to moderate knee osteoarthritis [J]. Journal of bodywork and movement therapies, 2021, 27: 597-604.

[26] Amani M, Shafizadegan Z, Taheri N. Effects of Dry Needling on Pain in Patients with Knee Osteoarthritis: A Preliminary Study [J]. Advanced biomedical research, 2022, 11: 47.

[27] Pang J C Y, Fu A S N, Lam S K H, et al. Ultrasound-guided dry needling versus traditional dry needling for patients with knee osteoarthritis: A double-blind randomized controlled trial [J]. PloS one, 2022, 17(9): e0274990.

[28] Wang X, Sun Q, Wang M, et al. Electrical Dry Needling Plus Corticosteroid Injection for Osteoarthritis of the Knee: A Randomized Controlled Trial [J]. Archives of physical medicine and rehabilitation, 2022, 103(5): 858-66.

**Table S3. Outcome measures**

|  | **Study** | **Outcome measures** |
| --- | --- | --- |
| 1 | Witt 2005 | WOMAC Index, WOMAC Pain, WOMAC Stiffness, WOMAC Physical function, Disability (PDI), Physical health (SF–36), Mental health (SF–36), Pain affective (SES, t standard scores), Pain sensoric (SES, t standard scores), Depression (ADS, t standard scores), Days with limited function, Days with pain in week 8 (diary), Days with medication in weeks 5–8 (diary) |
| 2 | Scharf 2006 | Success rate, Total WOMAC score, SF-12 physical subscale, SF-12 mental subscale, Global patient assessment, WOMAC pain, WOMAC stiffness, WOMAC functionality, von Korff classification |
| 3 | Foster 2007 | WOMAC Pain, WOMAC function, response rate, Knee pain intensity and unpleasantness (11 point NRS), severity of main  problem, Arthritis Self-Efficacy Scale, satisfaction with care |
| 4 | Chen 2013 | Success Rate, WOMAC Total Score, WOMAC Pain, WOMAC Function, WOMAC Stiffness, BPI Average Pain, SF-36 Physical Subscale, SF-36 Mental subscale, 6 Minute Walk Test (in feet), Patient Global Assessment |
| 5 | Hinman 2014 | Overall pain (11 point NRS), WOMAC function, WOMAC pain, Pain on walking (11 point NRS), Pain on standing (11 point NRS), Activity restriction (11 point NRS), AQoL-6D, SF-12 PCS, SF-12 MCS |
| 6 | Lin 2018 | Response rate, WOMAC pain, WOMAC function, VAS score, Physical health (SF-12), Mental health (SF-12) |
| 7 | Tu 2021 | Response rate, NRS, WOMAC pain, WOMAC function, WOMAC stiffness, SF-12 Physical health, SF-12 Mental health |
| 8 | Liu 2022 | WOMAC total score, WOMAC pain, WOMAC function, WOMAC stiffness, SF-12 PCS, SF-12 MCS, Knee flexion ROM, Knee extension ROM, Knee internal rotation ROM, Knee external rotation ROM |
| 9 | Sánchez Romero 2020 | Pain (NPRS), WOMAC pain, WOMAC function, WOMAC stiffness, WOMAC total score, EQ-5D, Barthel Index, Timed Up and Go Test, GROC, Consumption of medication, Falls rate, Mini-Mental State Examination |
| 10 | Ma 2023 | NPRS, WOMAC pain, WOMAC function, WOMAC stiffness, WOMAC total score, ROM |

**Table S4. Details of acupuncture treatment**

| **Study** | **Acupuncturist** | **Acupoints formula** | **Needle manipulation** |
| --- | --- | --- | --- |
| Witt 2005 | Specialized physicians who were trained (at least 140 h) and experienced in acupuncture. | At least six local acupuncture points from the following selection: ST34, ST35, ST36; SP9, SP10; BL40; KI10; GB33, GB34; LR8; EX-LE2, Xiyan. At least two distant points from the following selection: SP4, SP5, SP6; ST 6; BL20, BL57, BL58, BL60, BL62; KI3. | Sterile disposable one-time needles; physicians were able to choose the needle length and diameter. De qi was achieved (if possible); needles were stimulated manually at least once during each session. |
| Scharf 2006 | Practitioners with at least 2 years' experience in acupuncture. | Obligatory acupoints: Unilateral: ST34, ST36, Xiyan, SP9, SP10, GB34. Optional: Unilateral: 1 to 4 Ashi points. These Ashi points may be equivalent to the following local acupoints: LR7, LR8, KI10, BL40, GB33. Bilateral: 1 to 2 of 16 defined distant points (LI4, LI10, LR3, ST44, ST40, BL23, BL60, SP5, SP6, KI3, KI7, LI15, SI10, SI8, TE14, LU6). | Sterilized disposable steel needles, 30 × 0.3 mm. depth 0.5 to 3.5 cm according to the localization of points. Elicitation of De qi was attempted, followed by a manual stimulation of the needle, which has to be repeated twice. If both knees are affected, both were treated. |
| Foster 2007 | Physiotherapists trained in acupuncture to at least minimum national standards for membership of the Acupuncture Association of Chartered Physiotherapists. Two thirds of the physiotherapists had been qualified for more than 10 years and over half had been using acupuncture for more than three years. | 6 to 10 acupoints from 16 commonly used local and distal points were selected. Local points: SP9, SP10, ST34, ST35, ST36, Xiyan, GB34, and trigger points. Distal points: LI4, TH5, SP6, LI3, ST44, KI3, BI60, and GB41. | Sterilized disposable steel needles (30 × 0.3 mm) ; depth of insertion 5 to 25 mm. De qi sensation was elicited. |
| Chen 2013 | Fully trained and licensed acupuncturists | Primary acupoints: GB34, SP9, ST36, ST35 and Xiyan. Distal points: UB60, GB39, SP6, and KI3. 9 points in total. The same points were used for each affected leg. | Insertion depth 0.2 to 3 cm depending on the location of the point and patient's body size. The needles were left in place for 20 minutes, with a brief manipulation at the beginning and end of the treatment. The de qi sensation was not required and not specifically recorded. |
| Hinman 2014 | Family physicians registered as acupuncturists (mean, 33.3 years of clinical practice and 19.6 years of acupuncture experience) . All were members of the Australian Medical Acupuncture College, had completed university-level acupuncture training, and were formally accredited (by examination and supervised clinical experience) and registered as medical practitioner acupuncturists by the Medical Board of Australia. | Local points: SP9, SP10, ST34, ST35, ST36, LR7, LR8, LR9, KI10, BL39, BL40, BL57, GB34, GB35, GB36, local extra points in the hamstring muscles. Distal points: ST40, LR3, SP6, GB41, BL60. Segmental points: BL21, BL22, BL23, GB30, GB31. Non-segmental and general points: Ear Knee point. DU20, LI11, GV14, BL11. Other points could be used at the acupuncturist's discretion depending on clinical examination (eg, site and causes of pain). Initial treatment permitted a maximum of 6 points (4 on the study limb and 2 additional points chosen per protocol). In subsequent treatments, points were added and varied as clinically indicated. | Single-use Seirin needles (0.25 × 40 mm) |
| Lin 2018 | All acupuncturists were state-licensed and had at least five years of clinical experience. | 5 or 6 acupoints from 10 commonly used local points (ST34, ST35, ST36, EX-LE2,EX-LE5, GB33, GB34, SP9, SP10, LR8) ; 3 or 4 acupoints from 11 distal points (GB31, GB36, GB39, GB41, ST40, ST41, LR3, BL60, SP6, KI3, LI4). 8 to 10 acupoints in total (unilateral). | 0.30mm×40mm (for local points) and 0.30mm×25mm (for distal points) acupuncture needles; insertion depth10 to 30 mm depending on point location. Patients were treated with manipulations of twirling, lifting and thrusting on the basis of traditional Chinese acupuncture theory by acupuncturists. De qi was supposed to be achieved and needles will be stimulated manually at least 10 seconds. |
| Tu 2021 | Registered acupuncturists (11.7 ± 4.9 years of experience) performed the procedures. All acupuncturists were trained in standardized operating procedures prior to the start of the study. | 5 obligatory acupoints and 3 adjunct acupoints were used. Obligatory acupoints: ST35, EX-LE5, LR8, GB33 and an Ashi point (the point where the participant felt the most pain). Adjunct acupoints: ST32, ST34, EX-LE2, ST36, and ST40 for Yangming Meridian Syndrome; SP10, LR3, LR7, SP9, SP6, K13, SP4 and KI10 for Three-Yin Meridian Syndrome; BL39, BL40, BL57 and BL60 for Taiyang Meridian Syndrome; GB31, GB34, GB36, GB39 and GB41 for Shaoyang Meridian Syndrome. | Disposable sterile needles (0.25 mm × 25-40 mm), and HANS-200 electro-acupuncture devices were used. De qi was required. |
| Liu 2022 | The acupuncturists were specialists in Traditional Chinese Medicine at the hospitals, received specialized acupuncture training and licensed with at least 3 years of clinical experience. | The acupoints included EX-LE2, EX-LE4, ST35, SP10, ST34, SP9, GB34, ST36, BL40, KI10, LR7, LR8, BL39, and Ashi points. The 5 points with lowest PT (pain threshold) were identified as lower PT acupoints (corresponding to LPT group), and the 5 with the highest PT as higher PT acupoints (corresponding to HPT group). Patients with unilateral KOA were treated on the affected side. Patients with bilateral KOA were treated and assessed on their most painful side, and the non-trial affected low limbs were provided with acupuncture treatment on ST35, EX-LE4, GB34, ST36 and SP10. | Sterile single-use needles (0.30 × 40 mm); inserted vertically into the acupoints with a depth of 15 to 30 mm. The stimulation was performed with lifting and thrusting combined with rotating to elicit de qi. Needle manipulation was performed every 15 min. |

**
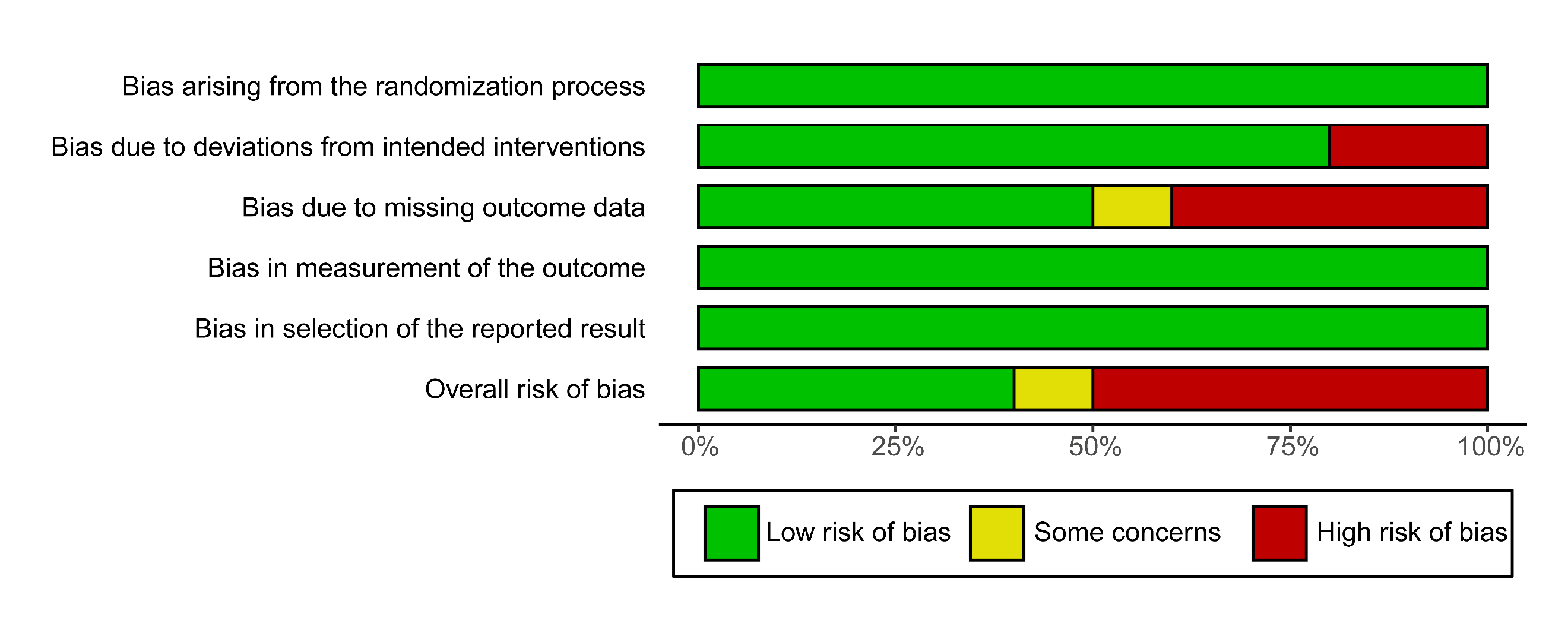
**

**Fig.S1** Risk of bias graph.

**
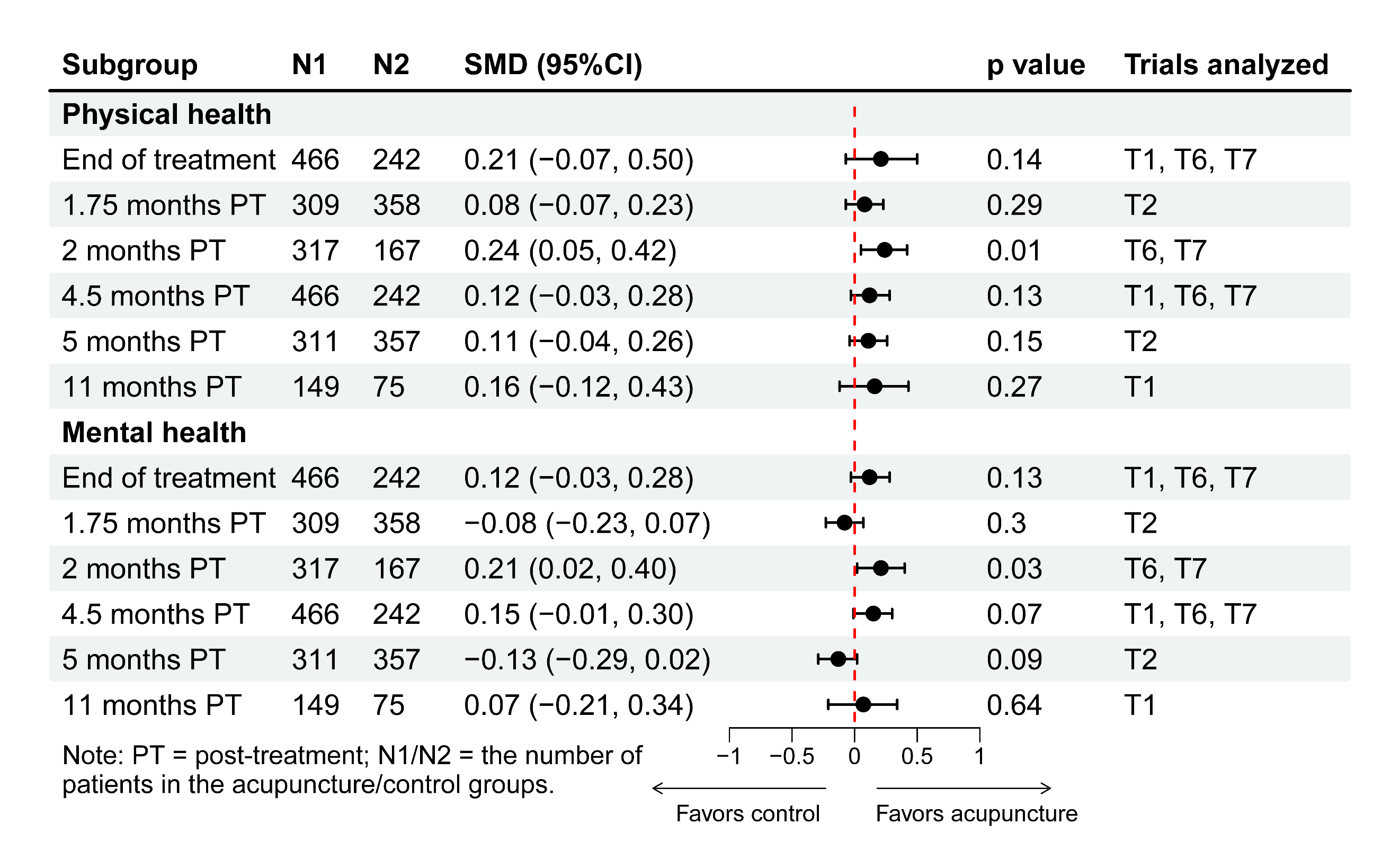
**

**Fig.S2.** Acupuncture versus sham acupuncture: Changes from baseline in physical health and mental health.

**
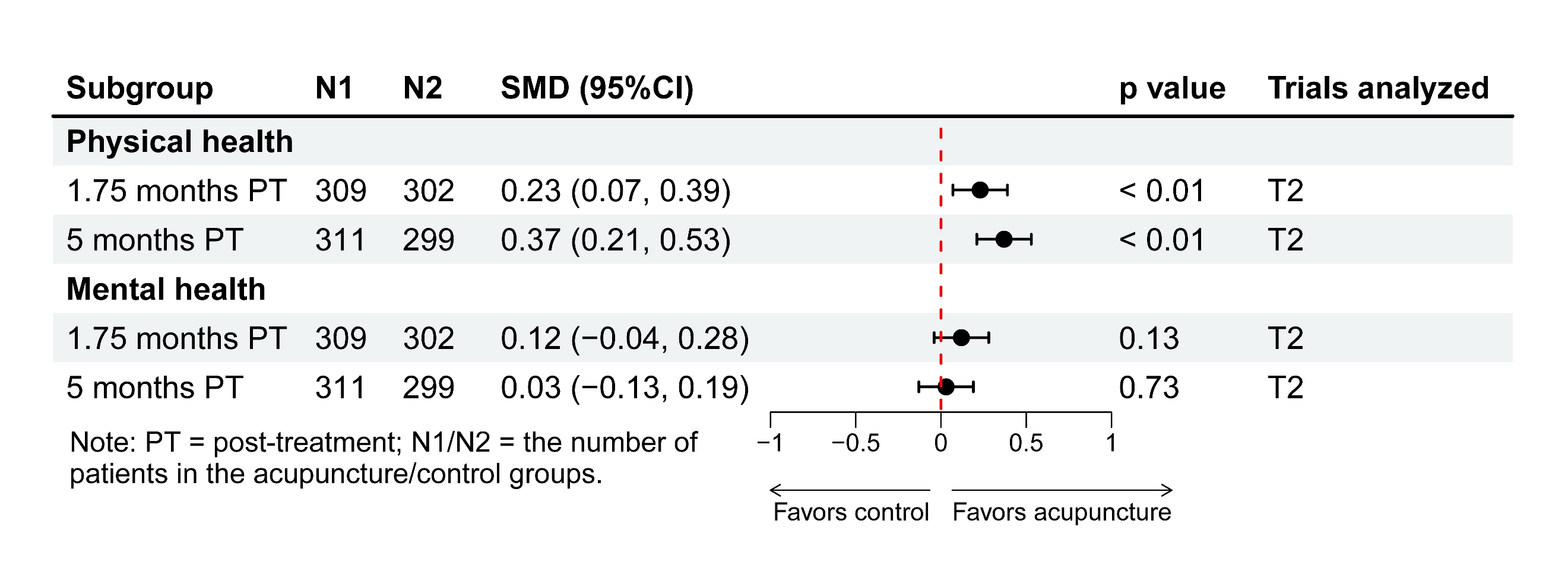
**

**Fig.S3**. Acupuncture versus usual care: Changes from baseline in physical health and mental health.

**
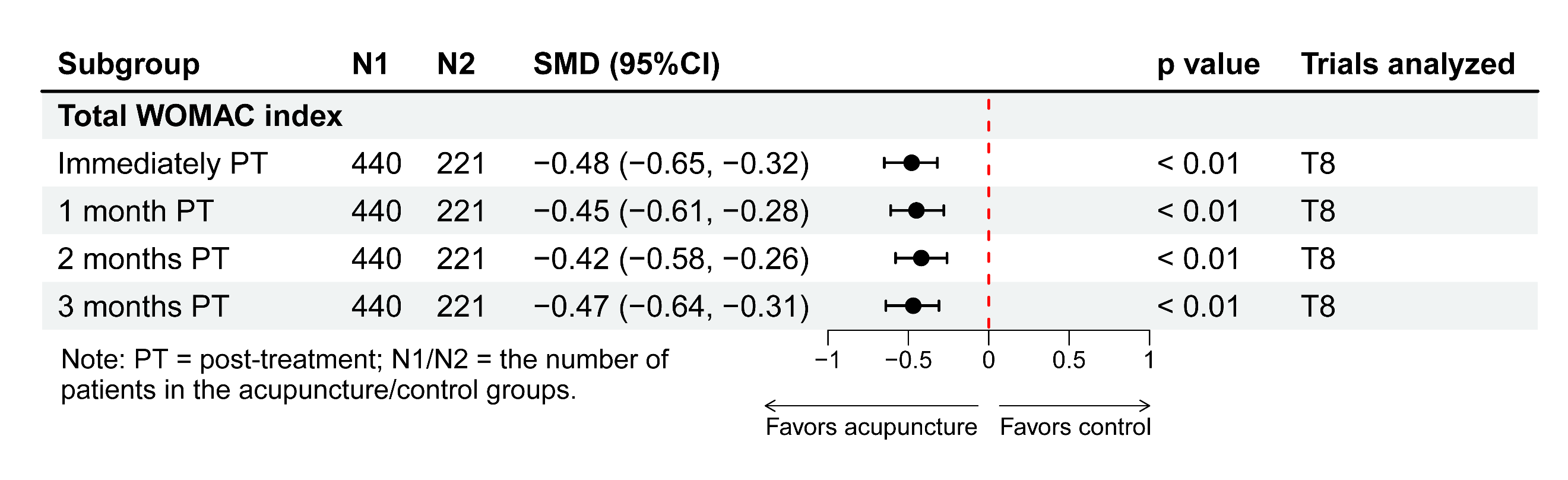
**

**Fig.S4**. Acupuncture versus no treatment: Changes from baseline in total WOMAC index.

**
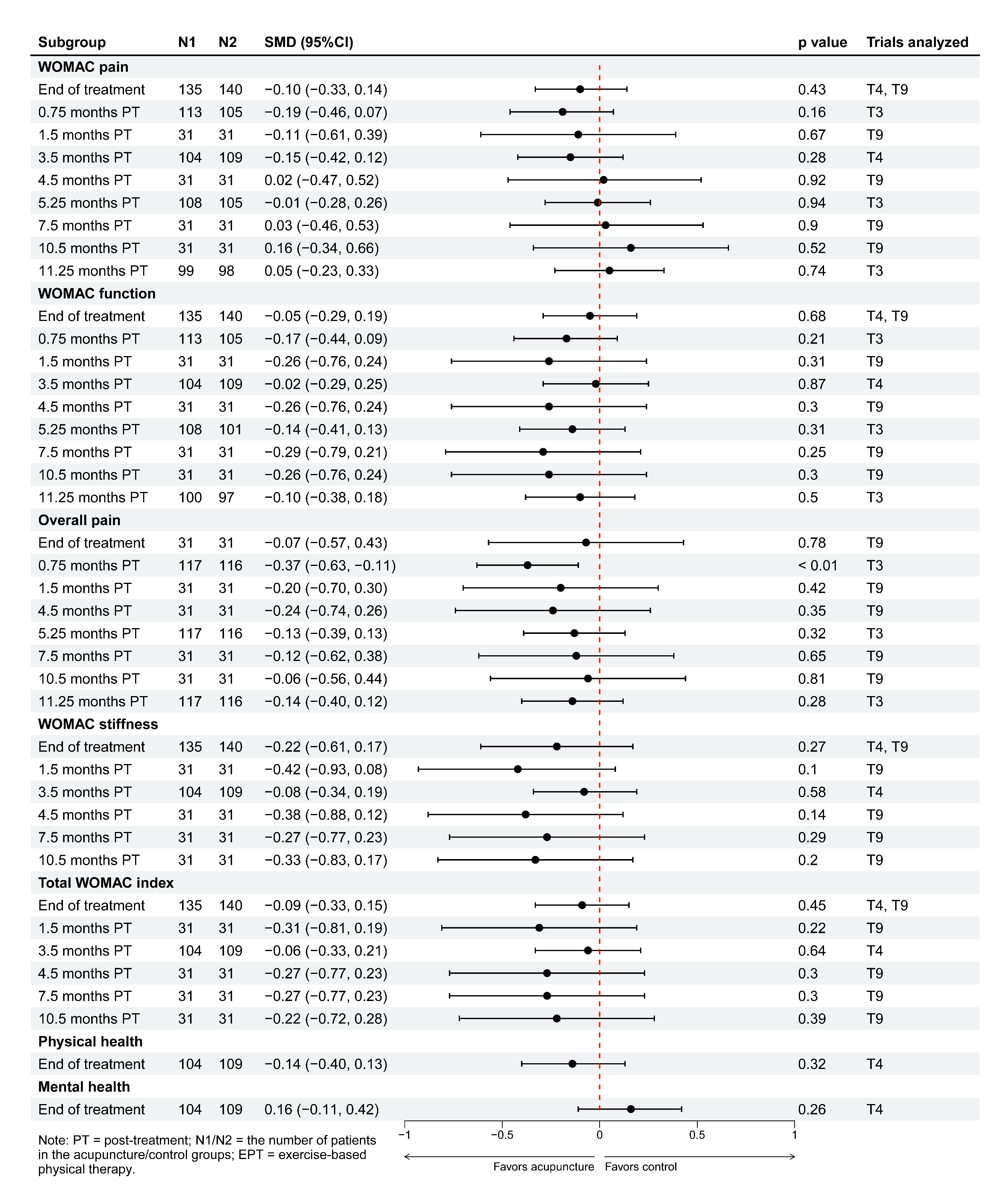
**

**Fig.S5**. Acupuncture plus EPT versus sham acupuncture plus EPT: Changes from baseline in WOMAC pain, WOMAC function, overall pain, WOMAC stiffness, and total WOMAC index.

**
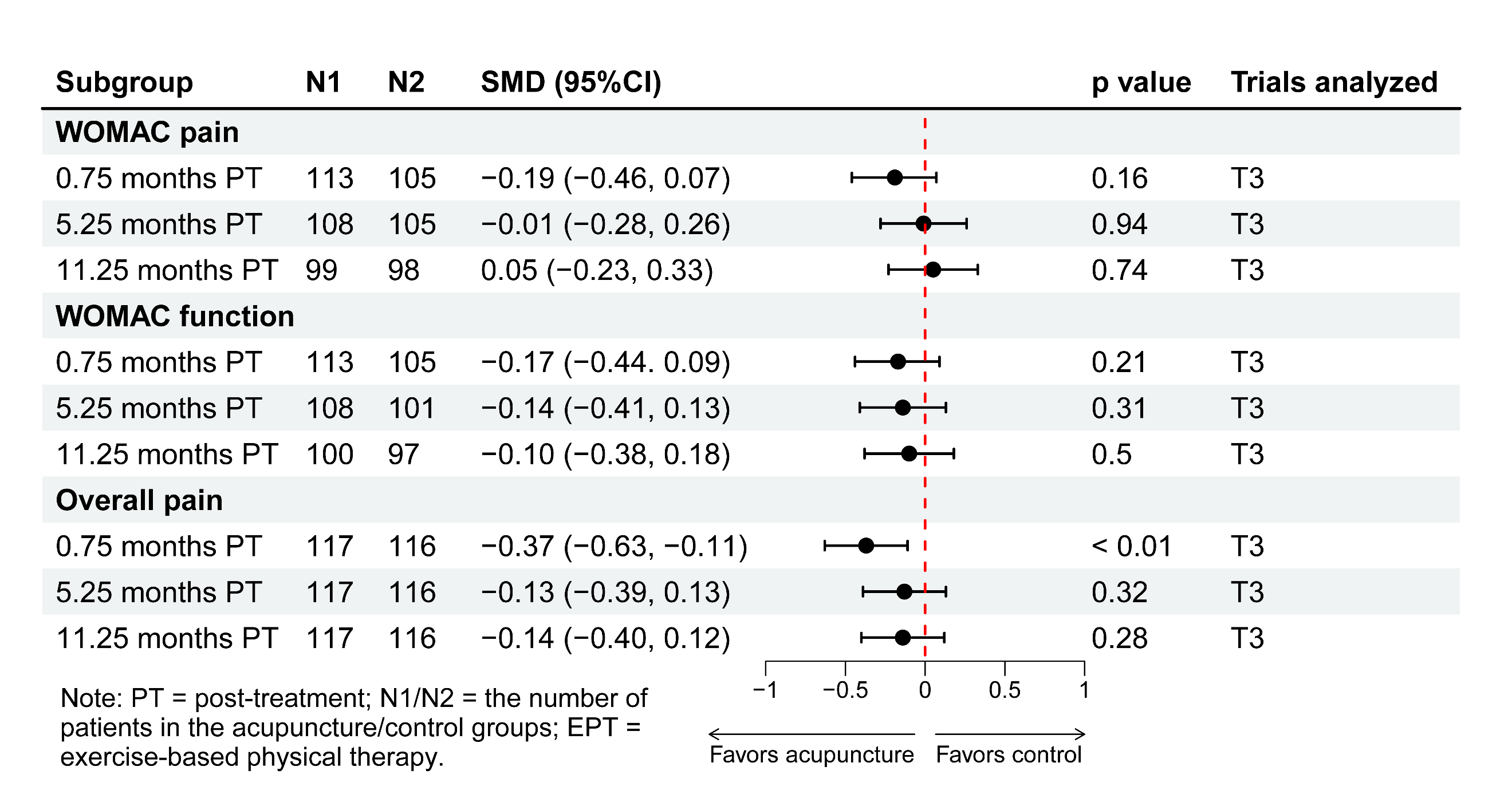
**

**Fig.S6**. Acupuncture plus EPT versus EPT: Changes from baseline in WOMAC pain, WOMAC function, overall pain.


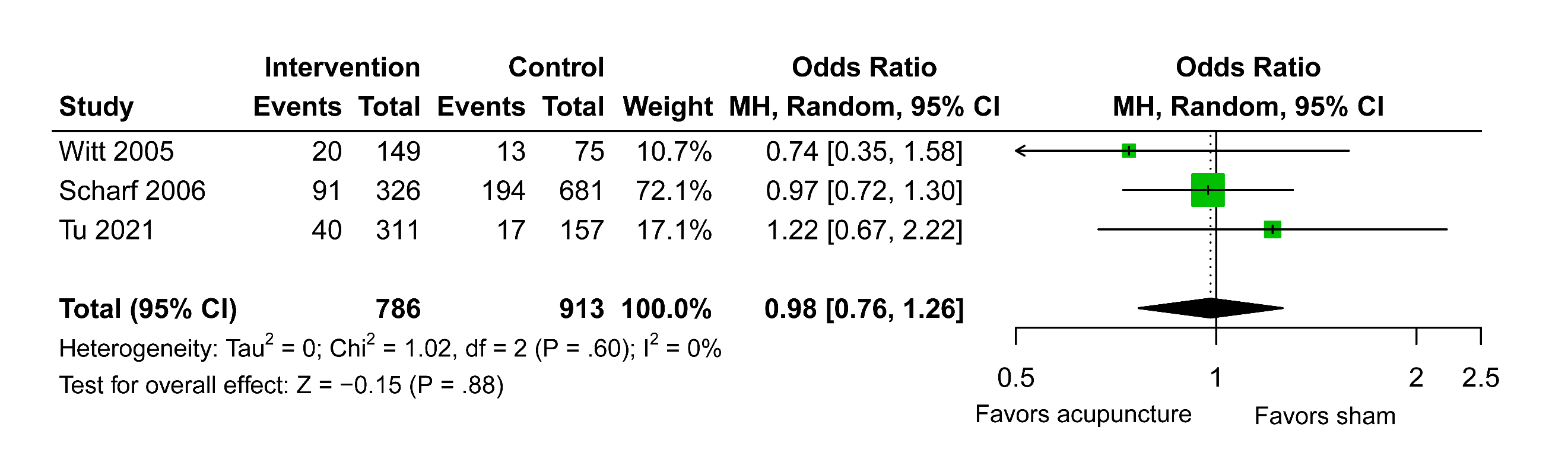


**Fig.S7**. Adverse events.

| **Section and Topic** | **Item #** | **Checklist item** | **Location where item is reported** |
| --- | --- | --- | --- |
| **TITLE** | | |  |
| Title | 1 | Identify the report as a systematic review. | 1 |
| **ABSTRACT** | | |  |
| Abstract | 2 | See the PRISMA 2020 for Abstracts checklist. | 1 |
| **INTRODUCTION** | | |  |
| Rationale | 3 | Describe the rationale for the review in the context of existing knowledge. | 2-3 |
| Objectives | 4 | Provide an explicit statement of the objective(s) or question(s) the review addresses. | 3-4 |
| **METHODS** | | |  |
| Eligibility criteria | 5 | Specify the inclusion and exclusion criteria for the review and how studies were grouped for the syntheses. | 5 |
| Information sources | 6 | Specify all databases, registers, websites, organisations, reference lists and other sources searched or consulted to identify studies. Specify the date when each source was last searched or consulted. | 4-5 |
| Search strategy | 7 | Present the full search strategies for all databases, registers and websites, including any filters and limits used. | Table S1 |
| Selection process | 8 | Specify the methods used to decide whether a study met the inclusion criteria of the review, including how many reviewers screened each record and each report retrieved, whether they worked independently, and if applicable, details of automation tools used in the process. | 6 |
| Data collection process | 9 | Specify the methods used to collect data from reports, including how many reviewers collected data from each report, whether they worked independently, any processes for obtaining or confirming data from study investigators, and if applicable, details of automation tools used in the process. | 6 |
| Data items | 10a | List and define all outcomes for which data were sought. Specify whether all results that were compatible with each outcome domain in each study were sought (e.g. for all measures, time points, analyses), and if not, the methods used to decide which results to collect. | 6-7 |
|  | 10b | List and define all other variables for which data were sought (e.g. participant and intervention characteristics, funding sources). Describe any assumptions made about any missing or unclear information. | 6-7 |
| Study risk of bias assessment | 11 | Specify the methods used to assess risk of bias in the included studies, including details of the tool(s) used, how many reviewers assessed each study and whether they worked independently, and if applicable, details of automation tools used in the process. | 7 |
| Effect measures | 12 | Specify for each outcome the effect measure(s) (e.g. risk ratio, mean difference) used in the synthesis or presentation of results. | 8 |
| Synthesis methods | 13a | Describe the processes used to decide which studies were eligible for each synthesis (e.g. tabulating the study intervention characteristics and comparing against the planned groups for each synthesis (item #5)). | 8 |
|  | 13b | Describe any methods required to prepare the data for presentation or synthesis, such as handling of missing summary statistics, or data conversions. | 8 |
|  | 13c | Describe any methods used to tabulate or visually display results of individual studies and syntheses. | 8 |
|  | 13d | Describe any methods used to synthesize results and provide a rationale for the choice(s). If meta-analysis was performed, describe the model(s), method(s) to identify the presence and extent of statistical heterogeneity, and software package(s) used. | 8 |
|  | 13e | Describe any methods used to explore possible causes of heterogeneity among study results (e.g. subgroup analysis, meta-regression). | NA |
|  | 13f | Describe any sensitivity analyses conducted to assess robustness of the synthesized results. | NA |
| Reporting bias assessment | 14 | Describe any methods used to assess risk of bias due to missing results in a synthesis (arising from reporting biases). | 7 |
| Certainty assessment | 15 | Describe any methods used to assess certainty (or confidence) in the body of evidence for an outcome. | NA |
| **RESULTS** | | |  |
| Study selection | 16a | Describe the results of the search and selection process, from the number of records identified in the search to the number of studies included in the review, ideally using a flow diagram. | 5, Fig.1 |
|  | 16b | Cite studies that might appear to meet the inclusion criteria, but which were excluded, and explain why they were excluded. | Table S2 |
| Study characteristics | 17 | Cite each included study and present its characteristics. | 8-9 |
| Risk of bias in studies | 18 | Present assessments of risk of bias for each included study. | 11-12 |
| Results of individual studies | 19 | For all outcomes, present, for each study: (a) summary statistics for each group (where appropriate) and (b) an effect estimate and its precision (e.g. confidence/credible interval), ideally using structured tables or plots. | Fig.3-6, Fig,S2-7 |
| Results of syntheses | 20a | For each synthesis, briefly summarise the characteristics and risk of bias among contributing studies. | NA |
|  | 20b | Present results of all statistical syntheses conducted. If meta-analysis was done, present for each the summary estimate and its precision (e.g. confidence/credible interval) and measures of statistical heterogeneity. If comparing groups, describe the direction of the effect. | 12-17 |
|  | 20c | Present results of all investigations of possible causes of heterogeneity among study results. | NA |
|  | 20d | Present results of all sensitivity analyses conducted to assess the robustness of the synthesized results. | NA |
| Reporting biases | 21 | Present assessments of risk of bias due to missing results (arising from reporting biases) for each synthesis assessed. | NA |
| Certainty of evidence | 22 | Present assessments of certainty (or confidence) in the body of evidence for each outcome assessed. | NA |
| **DISCUSSION** | | |  |
| Discussion | 23a | Provide a general interpretation of the results in the context of other evidence. | 17-18 |
|  | 23b | Discuss any limitations of the evidence included in the review. | 20 |
|  | 23c | Discuss any limitations of the review processes used. | 20 |
|  | 23d | Discuss implications of the results for practice, policy, and future research. | 19 |
| **OTHER INFORMATION** | | |  |
| Registration and protocol | 24a | Provide registration information for the review, including register name and registration number, or state that the review was not registered. | 4 |
|  | 24b | Indicate where the review protocol can be accessed, or state that a protocol was not prepared. | NA |
|  | 24c | Describe and explain any amendments to information provided at registration or in the protocol. | NA |
| Support | 25 | Describe sources of financial or non-financial support for the review, and the role of the funders or sponsors in the review. | 20 |
| Competing interests | 26 | Declare any competing interests of review authors. | 20 |
| Availability of data, code and other materials | 27 | Report which of the following are publicly available and where they can be found: template data collection forms; data extracted from included studies; data used for all analyses; analytic code; any other materials used in the review. | 20 |
